# Effects of mono- and dual-frequency ultrasounds on structure and physicochemical properties of faba bean proteins

**DOI:** 10.1016/j.ultsonch.2024.107144

**Published:** 2024-11-06

**Authors:** Shuyang Wang, Song Miao, Mohammad Hassan Kamani, Eoin G. Murphy, Da-Wen Sun

**Affiliations:** a Food Chemistry and Technology Department, Teagasc Food Research Centre, Moorepark, Fermoy, County Cork, Ireland; b Food Refrigeration and Computerized Food Technology (FRCFT), Agriculture and Food Science Centre, University College Dublin, National University of Ireland, Belfield, Dublin 4, Ireland

**Keywords:** Faba bean protein, Ultrasound, Protein modification, Physiochemical properties, Structure

## Abstract

Faba bean proteins are currently viewed as promising animal protein alternatives. However, certain functional properties e.g. relatively low solubility compared to whey protein isolates, can limit the application of faba bean protein isolates (FPIs) in certain food products. Therefore, it may be desirable to use modification approaches such as the application of ultrasound to alter such limiting physicochemical properties. In this study, Faba Bean Protein Isolates (FPIs) were treated by ultrasound with different frequencies (20 kHz, 40 kHz and 20 + 40 kHz) prior to hydration (1 %) at different pH levels (3, 7, and 9). Then the structure and physicochemical properties (i.e. particle size, ζ-potential, surface hydrophobicity, thermal behavior, and solubility) of control and untreated FPIs were investigated. Ultrasound treatment had no obvious effect on the molecular weight of FPIs, whereas it changed the secondary structure of FPIs from a more ordered structure to a more disordered structure. The applied treatment resulted in an increase in surface hydrophobicity across all treatment levels and pHs. It also decreased the particle size of FPI at pH 3, while it increased the particle size at pH 7 and 9, compared to the untreated FPI. In addition, the solubility and thermal properties of FPI were modified through the ultrasound treatment. The higher solubility of FPI could improve its potential to be used as a functional ingredient for many food applications. Ultrasound treatment at 20 kHz and 20 + 40 kHz had more effects on the physiochemical properties of FPI compared to that at 40 kHz. Overall, ultrasound treatment with different frequencies (20 kHz, 40 kHz, and 20 + 40 kHz) modified the structure and physiochemical properties of FPI to different degrees and may be beneficial for the development of FPI for certain food applications.

## Highlights

• Ultrasound treatment changed the secondary structure of FPI from a more ordered structure to a more disordered structure.
• Ultrasound treatment resulted in an increase in surface hydrophobicity across all treatment levels and pHs.
• 20 kHz ultrasound treatment had a highest improvement in FPI solubility at pH 3 and 7.
• The denaturation temperature of FPI treated by 20 kHz ultrasound increased at pH 3 and 7.
• Ultrasound treatment with 20 and 20+40 kHz has more modification in physiochemical properties of FPI than 40 kHz.

## Graphical abstract

**Figure:**
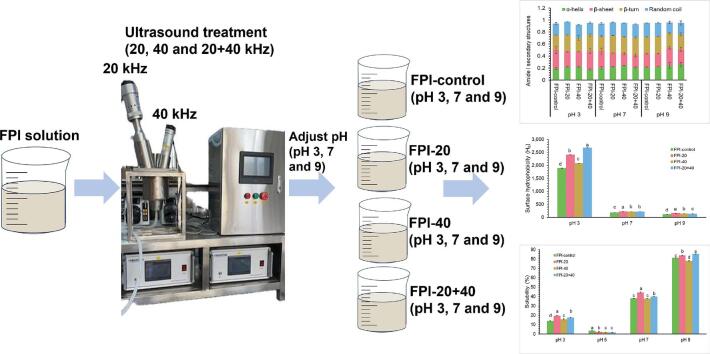

## 1 Introduction

Plant-based proteins are good alternatives to animal-based proteins, which are viewed to be favourable from environmental and sustainability perspectives [1], [2]. Legumes are an important source of plant-based proteins, due to their relatively high protein concentration [3]. Furthermore, cultivating legumes can help reduce greenhouse gases, sequester carbon, improve soil health, and have low water requirements [4]. In recent years, many studies have focused on legume proteins, such as soy proteins, pea proteins, lupin proteins, and so on [5]. However, the research on faba bean protein is limited, despite its potential.

From an agronomic perspective, faba bean presents better biological nitrogen fixation ability compared to other legumes and excellent environmental ability, which benefit the increase of subsequent crop yields and the reduction of environmental footprints [6]. The protein content of faba bean is 36–39 % [7]. Globulins and albumin are the main storage proteins in faba beans and are largely responsible for the rheological and textural properties of proteins [8], [9]. However, like other plant-based proteins, faba bean proteins have some limitations in their physicochemical functional properties, like denaturation temperature, solubility, emulsion properties, and gelling properties, which can influence certain applications within the food industry e.g. beverages where high solubility is required [6]. Therefore, in such cases, protein modification methods to alter the molecular structure or chemical groups of the proteins are required.

Ultrasound treatment is a non-thermal, sustainable, and novel technology for protein modification [10], [11]. Compared to the conventional thermal methods e.g. heat treatment, it is a more cost-effective method with less energy consumption [11]. High-frequency, low-intensity ultrasound (100 kHz-1 MHz, power < 1 W/cm^2^) is commonly utilized for protein modification [12], [13]. Ultrasonic treatment converts electricity into cavitational forces generated by pressure waves during a brief period [11]. This process leads to hydrodynamic shearing, which causes numerous stresses on materials. In the case of proteins, these stresses can have effects on their particle size and structure [14], [2]. In the research of Martínez-Velasco et al. [15], ultrasound treatment changed the secondary structure of faba bean proteins and improved the foaming properties. The combination of alkaline shifting and ultrasound treatment may also improve the functional properties of faba bean proteins, including solubility, emulsion properties, and foaming properties [16].

Ultrasound frequency is an important factor influencing cavitation intensity [17]. Compared to mono-frequency ultrasound, dual-frequency ultrasound tends to improve the cavitation activity due to higher shear force and bubble temperature, leading to more structure changes in proteins [18]. However, previous studies on ultrasound treatment of faba bean proteins have primarily examined the impact of mono-frequency at 20 kHz [15], [19]. There is limited literature concerning the effects of varying ultrasound frequencies on the properties of faba bean proteins.

In addition, pH conditions can affect the structure and size distribution of faba bean proteins. The structure and physicochemical properties of faba bean proteins shown in different pH conditions can affect their potential application in food products.

Therefore, the purpose of this study was to evaluate the effects of ultrasound treatment with 20 kHz, 40 kHz, and simultaneous modes 20 and 40 kHz on the structure and physicochemical properties of faba bean protein isolates (FPIs). Moreover, the structure and physicochemical properties of ultrasonicated FPIs at different pH conditions are also investigated to evaluate the combined effect of ultrasound treatment and pH. The study of the structure and physicochemical properties of modified FPIs would be useful for further research in FPI-based delivery systems and their application in food products.

## 2 Materials and methods

### 2.1 Materials

Dehulled faba beans were obtained from Askew & Barrett Pulses Ltd., Wisbech, UK. The grains were packed in polyethylene bags until required. All chemicals used in this study were analytical grade.

### 2.2 Faba bean proteins extraction

FPIs were prepared by alkaline solubilization and acid precipitation. Firstly, dehulled faba beans were milled into flour using a Retschgrinder (Model ZM200, Haan, Germany). The dehulled faba bean flour was dispersed into distilled water with a 1:10 ratio of flour and water. Then, the mixture was adjusted to pH 11 using 1  mol/l NaOH and stirred for 90 mins at the temperature of 45 ℃. After this, it was centrifuged at 9,000 g for 10  min at room temperature. The supernatant was collected and adjusted to pH 4.3 using 1  mol/l HCl to precipitate the proteins. Next, the precipitated proteins were collected by centrifugation at 9,000 g for 20  min and neutralized. Then, it was freeze-dried, and milled to obtain the dry protein powders, which were stored at 4 °C before analysis. After the extraction, the crude protein content was estimated using the Dumas combustion method (LECO Instruments, St. Joseph, MI, USA), with a nitrogen-to-protein conversion factor of 6.25 [16], [20].

### 2.3 Ultrasound treatment

The dual-frequency power ultrasound (Jiangsu University) is equipped with two frequency generators – 20 and 40 kHz. The maximum output acoustic power of 20 and 40 kHz generators was 800 and 400 W, respectively. There are two frequency modes: mono-frequency ultrasound (MFU) and dual-frequency ultrasound (DFU). The FPI samples were dispersed into distilled water at the concentration of 10 mg/ml and stirred overnight at 4 °C. 1400 mL aliquots of FPI suspensions were poured into the reaction vessel and circulated by a liquid circulating pump with a speed of 180 rpm. The two ultrasonic probes were submerged in the solution at a depth of 2.0 cm. The ultrasound treatment time was 15 min, and the temperature was controlled constantly by a chamber around the vessel containing thermostat-controlled recycling water. The sweep cycles and pulse ratio (on-time/off-time) were set to 5  s on-time and 2  s off-time (5  s/2 s). The frequencies selected were i) 20 kHz (MFU), ii) 40 kHz (MFU), and iii) 20 and 40 kHz (DFU). After treatment, the samples were stored at 4 °C before analysis. The samples were coded as FPI-20, FPI-40, and FPI-20 + 40, corresponding to the FPI sample prepared by ultrasound with the frequency of 20 kHz, 40 kHz, and simultaneous mode 20 and 40 kHz. The untreated FPI sample was dispersed into distilled water at the concentration of 10 mg/ml and was coded as FPI-control.

### 2.4 Structure properties

#### 2.4.1 Sodium dodecyl sulfate–polyacrylamide gel electrophoresis (SDS-PAGE)

SDS-PAGE of FPI samples was conducted using polyacrylamide gels. The pH values of the ultrasonic and control FPI samples were adjusted to 3, 7, and 9 using 1 M HCl or NaOH. Then, the solutions were centrifuged at 10,000 g for 15 min at room temperature. The supernatants were collected and protein content of them was diluted to 1 mg/mL, then mixed with NuPAGE LDS sample buffer (4X, Invitrogen, Thermo Fisher Scientific, Cork, Ireland) at a ratio of 3:1. For reducing condition, NuPAGE Reducing Agent (3 µL, 10X, Invitrogen, Thermo Fisher Scientific, Cork, Ireland) contained 500 mM dithiothreitol (DTT) were added to the samples. The mixtures were boiled in a water bath for 5 min at 90 °C and then centrifuged at 10,000 g for 5 min. After cooling to room temperature, 10 μL of each mixture was loaded onto the polyacrylamide gel (Bio-Rad Laboratories, Dublin, Ireland), composed of 12 % MINI-Protein® TGX™ precast polyacrylamide gels. The electrophoresis process was carried out at 120 V. After electrophoresis, the gel was stained with Coomassie Blue R250 and then washed with 10 % acetic acid and 20 % ethanol.

#### 2.4.2 Fourier transform infrared (FTIR) spectroscopy

FTIR spectra of the control and treated FPI were collected by FTIR spectroscopy (Bruker Tensor 27, Bruker Optik GmbH, Ettlingen, Germany) equipped with ATR cell (PIKE Technology Inc., Madison, WI, USA). The solutions of the control and treated FPIs with the pH values of 3, 5, 7, and 9 were freeze-dried and ground to gain the powders. Then, the sample was placed on the surface of the ATR crystal and pressed with a flat-tip plunger. The spectra were measured in the wavenumber range of 900 to 4000 cm^−1^ with an average of 120 scans at a resolution of 4 cm^−1^. Fourier self-deconvolution and second derivative analysis were performed, and then the peaks were fitted in the amide-I region (1700–1600  cm^−1^) band using the Peakfit 4.12 software (Jandel Scientific Software, San Rafael, CA, USA).

### 2.5 Physicochemical properties

#### 2.5.1 Particle size and ζ-potential

The Z-average size and ζ-potential were measured by a Zetasizer Nano ZS (Malvern Instruments Ltd., Worcestershire, UK) through the dynamic laser scattering (DLS) method at room temperature [16]. For ζ-potential measurement, the protein solutions (1 mg/mL) were adjusted to pH 3, 5, 7, and 9 using 1 M HCl or NaOH. For Z-average size measurement, the protein solutions were adjusted to pH 3, 7, and 9 using 1 M HCl or NaOH. Then, the solutions were centrifuged at 10,000 g for 15  min at room temperature. The supernatants were collected and diluted to 1 mg/mL for the Z-average size measurement. The refractive index of the zeta size is 1.45 and 1.33 for proteins and water, respectively.

#### 2.5.2 Surface hydrophobicity

The surface hydrophobicity (H_0_) of samples was measured by the fluorescence probe method of Kato and Nakai [21], adapted by Zhang et al. [22]. A 1-anilino-8-naphthalene-sulfonate (ANS) (8.0 mmol/L in 0.01 mol/L phosphate buffer, pH = 7.0) was selected as the fluorescence probe. The supernatants of protein solutions with pH 3, 7, and 9 were diluted to obtain serial concentrations of samples (0.2 to 1.0 mg/mL protein concentration). Then, the sample (3.0 mL) was mixed with ANS solution (30 μL). After keeping each sample for 15 min in the dark, the fluorescent intensity was measured by a Varian Cary Eclipse fluorescence spectrophotometer at the excitation wavelength of 390 nm and the emission wavelength of 470 nm [23]. The fluorescent intensity values for diluted protein blanks (without the ANS probe) were measured and subtracted from the values of protein solutions with ANS. H_0_ can be calculated by the slope of a straight line obtained from plotting fluorescence intensity against protein concentration.

#### 2.5.3 Differential scanning calorimetry

Differential scanning calorimetry (DSC) was measured by a DSC (TA Instruments, New Castle, DE, USA) following the approach of Boostani et al. [24] with slight adjustments. The solutions of the control and treated FPI were freeze-dried and ground to obtain powders. About 10 mg of 20 % w/w suspensions of the control and treated FPI, dispersed in the different buffers with the pH values of 3, 7, and 9, were added to aluminum pans. The aluminum pans were hermetically sealed and scanned from 20 to 150 °C at a heating rate of 5 °C /min in an inert nitrogen environment. An empty aluminum pan was used as the reference. Data was analyzed using the Advantage/Universal Analysis software (TA Instruments, New Castle, DE, USA).

#### 2.5.4 Solubility

Protein solubility was measured using the Kjeldahl method. The pH values of the control and treated FPI solutions were adjusted to 3, 5, 7, and 9 using 1  mol/L NaOH or HCl. The samples were then centrifuged at 10,000 g for 15 min at room temperature. The supernatants were collected, and the protein content was determined using the Kjeldahl method. The solubility (%) was calculated from the following equation:

(1) $$Solubility\left(\%\right)=\frac{\mathrm{Protein}\;\mathrm{content}\;\mathrm{of}\;\mathrm{supernant}}{\mathrm{Total}\;\mathrm{protein}\;\mathrm{content}\;\mathrm{of}\;\mathrm{the}\;\mathrm{sample}}\times 100$$

### 2.6 Statistical analysis

All data were performed as means and standard deviation (n = 3). Significantly differences (p < 0.05) in mean values were classed using Duncan’s mean comparison by SPSS 27.0 (SPSS Inc., Chicago, IL, USA).

## 3 Results and discussion

### 3.1 Sodium dodecyl sulfate–polyacrylamide gel electrophoresis (SDS-PAGE)

**Fig. 1:**
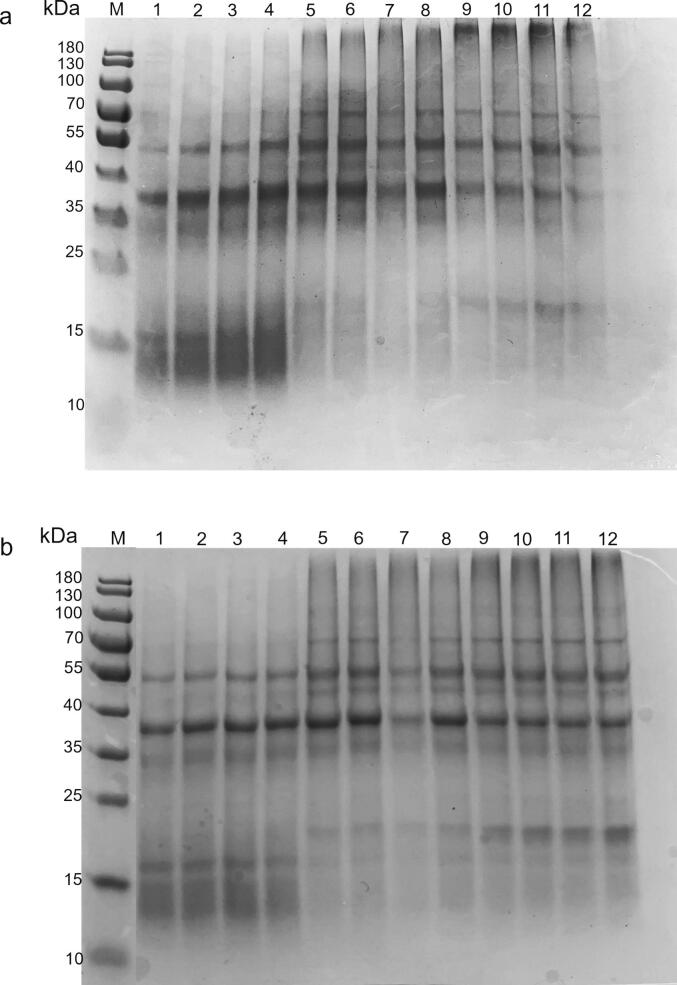
(a) Non-reducing and (b) reducing SDS-PAGE profiles of control and ultrasound-treated (20 kHz, 40 kHz, and 20 + 40 kHz) FPIs. Lane M: molecular weight standards, Lane 1–4: FPI-control, FPI-20, FPI-40, and FPI-20 + 40 at pH 3, Lane 5–8: FPI-control, FPI-20, FPI-40, and FPI-20 + 40 at pH 7, Lane 9–12: FPI-control, FPI-20, FPI-40, and FPI-20 + 40 at pH 9.

SDS-PAGE profiles of the control and treated FPI at the pH values 3, 7, and 9 are shown in Fig. 1. The components of faba bean proteins are mainly globulins with lower amounts of albumin, glutelin, and polyamine [25]. The globulins include 11S globulin (legumin) and 7S globulin (vicilin). 11S globulin (legumin) hexamers with an MW of approximately 300 to 370 kDa consist of six intermediary subunits with an MW of, on average, 50 to 60 kDa [26]. Each intermediary subunit consists of an acidic α subunit (∼36 kDa) and a basic β subunit (∼22 kDa), stabilized by intermolecular disulfide bonds [27]. 7S globulin (vicilin) is a trimeric protein containing several subunits (48–50 kDa each) with an average MW of 150 kDa, which accounts for ∼ 20 % of the total globulins [28], [29].

For the control and ultrasound-treated FPIs at pH 7, under non-reducing conditions, SDS-PAGE presented major polypeptide bands with molecular weights of approximately 53–55 and 36–38 kDa (Fig. 1a; lane 5, 6, 7, 8), which were attributable to legumin subunits. The legumin subunits were also shown at 70 kDa. However, the band was less dense than at 53–55 kDa. This corresponds to the results of Berrazaga et al. [26], in which the more intensive legumin subunits band was observed at 53–55 kDa. Besides, some vicilin fractions at about 48 kDa were observed in Fig. 1a with a less intensive band. According to Fig. 1b, under reducing conditions, the breakup of disulfide bonds resulted in the presence of acidic (∼35–36 kDa) and 2 basic (∼21 and 18 kDa, respectively) legumin polypeptides. There was no difference in the bands related to vicilin fraction under non-reducing and reducing conditions. It may be because vicilin proteins are devoid of sulfur-containing amino acids [19].

There was no obvious change in protein profiles of the FPI treated by ultrasound compared to the control FPI, while protein profiles of the FPI changed at different pHs. According to Fig. 1a, under non-reducing conditions, similar results were obtained for all samples at molecular weights 53–55 and 38 kDa. However, at pH 3, lower molecular weight bands (∼15 kDa) related to vicilin polypeptides were observed, along with lower amounts of the higher molecular weight bands (48 kDa and 70 kDa). The bands of the control and treated FPI at pH 9 (lanes 9, 10, 11, 12) showed more intense smears of high molecular weight aggregates on the border of the gel at pH 7. These bands were entirely absent from the pH 3 samples (lanes 1–4). Under reducing conditions, at pH 9, these large molecular weight aggregates were still observed. This suggests that other types of cross-linking can occur at alkaline pH besides disulfide bonds.

### 3.2 Fourier transform infrared (FTIR) spectroscopy

**Fig. 2:**
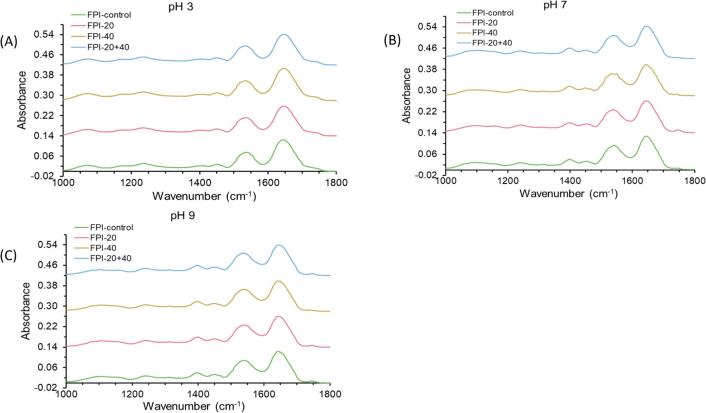
FTIR infrared spectra in the amide vibrational region for the control and ultrasound treated (20 kHz, 40 kHz, and 20 + 40 kHz) FPIs at pH 3, 7 and 9.

FTIR spectroscopy is a molecular vibrational spectroscopic technique widely used to characterize the secondary structure of proteins [17]. Fig. 2 shows the FTIR spectra of the control and ultrasound-treated FPIs at different pHs. Looking at the spectra of the control FPI, the main features were three intense bands corresponding to the amide I region (1700 to 1600  cm^−1^), amide II region (1550 to 1530  cm^−1^), and amide III region (1300 to 1200  cm^−1^). The amide-I band can be attributed to C = O stretching, while the amide-II band can be related to N-H stretching. The amide III band can be associated with C-N stretching and N-H bending [30]. In addition, the spectra showed another intense band with the wave number around 1400 cm^−1^. The band may be attributed to C–H bending caused by the deformational vibrations of the CH_2_ functional groups [31]. At pH 7, compared to the control FPI, ultrasound treatment with 20 kHz and 20 + 40 kHz reduced the areas associated with amide I and amide II. FPI-40 showed a decrease in band intensity in the three amide zones. At pH 3, FPI-40 presented an increase in band intensity in the three amide zones with respect to the control FPI. FPI-20 and FPI-20 + 40 showed a decrease intensity in the amide I and an increase intensity in the amide III. At pH 9, all the treated FPI showed a slight decrease in intensity in the amide I zone compared to the control FPI, while there is no obvious variation in the amide II and amide III zones.

The cavitation force produced by ultrasound treatment could lead to flexural vibration frequencies of the intra- and inter-molecular hydrogen bonds, which is related to C–O and N–H stretching [32]. It is hypothesized that ultrasound treatment with different frequencies had different ultrasonic, mechanical and chemical actions, which resulted in different disruptions of the interaction between the protein molecules and different changes in the internal structure of the protein molecule [33].

Analysis of the protein secondary structure is commonly based on amide I (1700–1600 cm^−1^) due to its sensitivity to the structure of the protein backbone [33]. After deconvolution and the overlapping components extraction procedures, a self-deconvolution and secondary derivative were fitted by a Gaussian function [34]. As per previous studies, the fitted peaks were assigned to β-sheet (1610–1637 cm^−1^), random coil (1638–1648 cm^−1^), α-helix (1649–1660 cm^−1^), β-turn (1660–1680 cm^−1^), and β-antiparallel sheet (1680–1692 cm^−1^) [35], [36]. Secondary structure contents of the control and ultrasound-treated FPI at different pH are shown in Table 1. The results showed that at pH values of 3 and 7, β-turn and random coil content of FPI treated by ultrasound increased compared to the control FPI, while a decrease of β-sheet content was displayed in the ultrasound-treated FPI. The β-turn and random coil can be assigned to disordered secondary structures, whereas β-sheet can be seen as ordered secondary structures [37]. The results suggested that the ultrasound treatment might promote the unfolding of protein structures. Ultrasound treatment with different intensities resulted in the variation of the secondary structure. In the ultrasound study on quinoa proteins, Luo et al. [37] reported similar results, in which β-turn and random coil increased, and β-sheet decreased after the ultrasound treatment. The study of Xu et al. [33] presented similar results. Ultrasound-treated casein showed a decrease in β-sheet and an increase in β-turn and random coil.

**Table 1:** Contents of the secondary structure components of the control and ultrasound-treated (20 kHz, 40 kHz, and 20 + 40 kHz) FPIs at pH 3, 7 and 9 from the spectral deconvolution of the amide Ⅰ band.

pH	Samples	Amide I secondary structures				
		β-sheet	Random coil	α-helix	β-turn	β-antiparallel sheet
pH 3	FPI-control	0.31 ± 0.06	0.19 ± 0.02	0.19 ± 0.02	0.25 ± 0.00	0.05 ± 0.02
	FPI-20	0.25 ± 0.00	0.22 ± 0.00	0.22 ± 0.00	0.28 ± 0.00	0.04 ± 0.01
	FPI-40	0.25 ± 0.00	0.22 ± 0.01	0.22 ± 0.01	0.23 ± 0.04	0.07 ± 0.03
	FPI-20 + 40	0.31 ± 0.04	0.20 ± 0.02	0.17 ± 0.02	0.27 ± 0.03	0.05 ± 0.01
pH 7	FPI-control	0.28 ± 0.05	0.21 ± 0.01	0.20 ± 0.02	0.25 ± 0.01	0.05 ± 0.00
	FPI-20	0.23 ± 0.00	0.22 ± 0.01	0.22 ± 0.00	0.29 ± 0.00	0.04 ± 0.01
	FPI-40	0.20 ± 0.02	0.23 ± 0.00	0.24 ± 0.00	0.28 ± 0.01	0.05 ± 0.02
	FPI-20 + 40	0.20 ± 0.02	0.22 ± 0.01	0.21 ± 0.01	0.30 ± 0.01	0.07 ± 0.03
pH 9	FPI-control	0.23 ± 0.01	0.23 ± 0.00	0.21 ± 0.01	0.28 ± 0.00	0.06 ± 0.00
	FPI-20	0.22 ± 0.00	0.22 ± 0.00	0.22 ± 0.00	0.29 ± 0.00	0.05 ± 0.01
	FPI-40	0.30 ± 0.02	0.18 ± 0.02	0.24 ± 0.05	0.24 ± 0.01	0.04 ± 0.00
	FPI-20 + 40	0.25 ± 0.03	0.19 ± 0.03	0.26 ± 0.03	0.25 ± 0.03	0.05 ± 0.01

However, at pH 9, there was an increase in α-helix and β-sheet and a decrease in random coil and β-turn of FPI treated by ultrasound with frequency 40 kHz and 20 + 40 kHz. The higher pH value could lead to the unfolding of protein structures. The combination effect of pH and ultrasound appears to alter the effect of ultrasound on secondary structure.

### 3.3 Particle size and ζ-potential

**Fig. 3:**
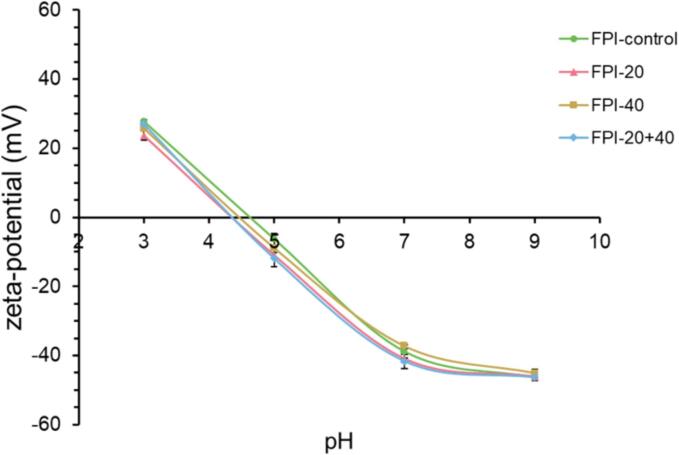
Zeta-potential-pH profile of the control and ultrasound-treated (20 kHz, 40 kHz, and 20 + 40 kHz) FPIs.

Zeta potential reflects the net charge. The ζ-potential values of the control and ultrasonic FPI samples are presented in Fig. 3. There was no significant change in the trend of the ζ-potential-pH profile of all samples. The ζ-potential shifted from positive to negative values when the pH moved from 3 to 9. The minimal ζ-potential of all FPI samples was at the pH values between 4 and 5, which means the isoelectric point (pI) range of FPI. The pI values of ultrasonic FPI samples were lower than the control FPI samples. FPI-20 had the lowest pI values.

Furthermore, the control FPI and the ultrasonic FPI samples showed similar ζ-potential values at pH 9. The surface charge of proteins is primarily related to the ionization of surface groups of the protein. Hence, it means that the treatments had less effect on the distribution of the ionizable amino acid residues on the faba bean proteins' surface at pH 9. The treated FPI samples showed higher negative charge values than the control FPI at pH 7 but lower values at pH 3. FPI-20 and FPI-20 + 40 presented more changes than FPI-40. The change in the surface charge can be caused by the balance of the exposure of non-polar hydrophobic residues and ionic groups, which is related to the different degrees of unfolding of the protein after the ultrasound treatment [15].

Table 2 shows the Z-average size of the control and treated FPI at pH values of 3, 7, and 9. Z-average size is an intensity-based measurement that emphasizes the larger particles in the distribution. Thus, Z-average size can act as a sensitive parameter for the presence of large aggregates in the FPI. The particle size of the FPI sample treated by ultrasound was slightly smaller than that of the control FPI at pH 3. It can be attributed to the shear forces generated by ultrasonic cavitation, which can disrupt hydrogen bonding and hydrophobic and electrostatic interactions in large protein aggregation [16]. However, the particle size of the FPI sample treated by ultrasound increased compared to the control FPI at pH 7 and 9. This result indicates ultrasound resulted in aggregation of faba bean proteins at higher pH [32]. In addition, pH was an important factor that affected the particle size of FPI. For the FPI-control, the minimum particle size was shown at pH 7, while the maximum particle size was presented at pH 9. Therefore, pH can influence the structure of FPI, which may lead to the reduction of electrostatic repulsion between neighboring molecules of protein isolates, promoting them to aggregate via hydrophobic and non-covalent interactions [38]. For the FPI-20 and FPI-20 + 40, the particle size increased with an increase in the pH of the solution, while FPI-40 showed the minimum particle size at pH 7. It can be attributed to the different degrees of structural changes of FPI through ultrasound treatment with different intensities. At pH 3 and 7, the FPI through ultrasound treatment with 20 and 20 + 40 kHz presented a greater change in the particle than that with 40 kHz.

**Table 2:** The particle size of the control and ultrasound-treated (20 kHz, 40 kHz, and 20 + 40 kHz) FPIs at pH 3, 7 and 9.

Samples	Particle size (nm)		
	pH3	pH7	pH9
FPI-control	121.3 ± 0.9^a^	94.35 ± 2.75^c^	185.57 ± 5.26^c^
FPI-20	116.95 ± 1.65^b^	119.4 ± 1.1^a^	195.87 ± 3.81^bc^
FPI-40	115.85 ± 0.45^b^	102.87 ± 1.97^b^	208.93 ± 5.35^ab^
FPI-20 + 40	117.25 ± 0.55^b^	117.55 ± 0.65^a^	210.07 ± 7.65^a^

Note: Values are given as mean ± standard deviation. Different lowercase letters in the same column are significantly different (p < 0.05).

### 3.4 Surface hydrophobicity

**Fig. 4:**
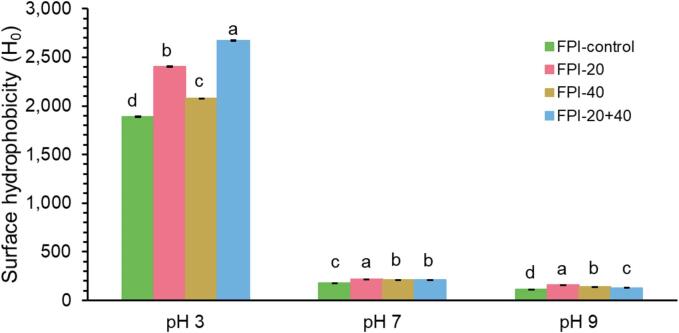
pH-dependent H0 of the control and ultrasound-treated (20 kHz, 40 kHz, and 20 + 40 kHz) FPIs. The different letters indicate that each sample has a significant difference (p < 0.05).

Surface hydrophobicity (H_0_) reflects the distribution of hydrophobic patches at the protein surface. It is important to protein stability and conformation and is further influenced the protein functional properties such as emulsifying and foaming properties [19]. Fig. 4 shows the H_0_ of the control FPI and ultrasonic FPI at the pH values of 3, 7, and 9. From the figure, H_0_ of the control and ultrasonic FPI decreased with increasing pH. This increase in surface hydrophobicity at pH 3 can be attributed to the structural change of FPI at acid pH [39]. The decrease in H_0_ at pH 7 and 9 may be due to the same negative charge carried by both the protein isolates and ANS probes. The net electrostatic repulsive forces could reduce the interaction between aromatic moieties on protein isolates with the ANS probe, leading to poorer estimates of the actual value [40]. Compared to FPI at pH 7, FPI formed larger protein aggregates at pH 9, potentially hiding some hydrophobic residues. These residues are not exposed to the surface and do not interact with the ANS probe [41].

The FPI treated by ultrasound tends to present higher H_0_ values than the control FPI at the three different pH values. This could be caused by the exposure of some hydrophobic residues. The ultrasonic cavitation promotes the disruption and rearrangement of some primary protein aggregates and molecules [42]. Thus, the buried hydrophobic residues could be exposed to the surface of the protein. The higher H_0_ increase of ultrasound-treated FPI at the pH value of 3 compared to the pH value of 7 and 9. The results of particle size showed that the treated FPIs had smaller particle sizes than the control FPI at pH 3. The combined effect of ultrasound treatment and pH could break down the large protein aggregates and extend the surface of proteins. More buried hydrophobic residues could be exposed to the protein surface.

The different frequencies of ultrasound treatment also affected the H_0_ of FPI. FPI-20 showed the highest H_0_ at pH 7 and 9, while FPI-20 + 40 presented the highest H_0_ at pH 3. FPI-20 and FPI-20 + 40 were treated by ultrasound with higher intensity than FPI-40. This could lead to more change in the structure of FPI and more relocation of hydrophobic residues.

### 3.5 Differential scanning calorimetry (DSC)

**Fig. 5:**
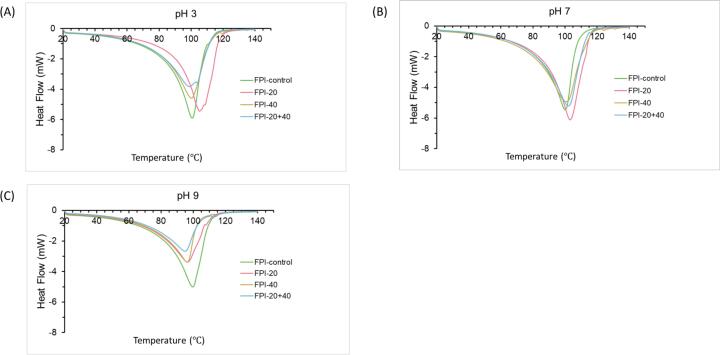
DSC thermograms of the control and ultrasound-treated (20 kHz, 40 kHz, and 20 + 40 kHz) FPIs at pH 3, 7 and 9.

DSC thermograms are shown in Fig. 5, where the treated and untreated faba bean protein suspensions were heated from 20 to 150 °C. The results indicate that the treated and untreated samples present a single endothermic peak varied from about 100.08 °C to 102.81 °C at pH 7. The melting peak means the denaturation of the faba bean protein. The result is a little higher than the results of Hall & Moraru [43], who reported a single endothermic peak at 94 °C. This could be caused by the differences in the preparation of faba bean samples. The faba bean proteins, after ultrasound treatment, presented a slightly higher peak temperature compared to the control proteins at pH 7, especially FPI-20. At other pHs, there was almost no difference in peak temperature of control samples (p < 0.05). However, the ultrasonic samples showed lower peak temperatures at pH 9 than at pH 7. FPI-20 + 40 treatment appeared to result in lower peak temperature compared to FPI-20 and FPI-40 treatment. Different trends were observed at pH 3; peak temperature increased in FPI-20 but decreased in FPI-20 + 40. There was no difference in FPI-40 compared to FPI-control.

Enthalpy refers to the heat energy consumed in the process of protein denaturation [44]. According to Table 3, the ΔH of the control proteins was higher than the ultrasonic proteins at pH 3 and 9. At these pHs, treatment at 20 + 40 kHz especially reduced ΔH. Overall, the results suggested that the ultrasound treatment decreased ΔH at acidic (pH 3) and alkaline (pH 9) conditions, and the ultrasound frequency influenced ΔH. The study of Lo et al. [44] showed a similar result, in which the ΔH decreased for lupin protein after ultrasound treatment. This indicated that faba bean protein is partially denatured [43]. However, ultrasonic samples showed higher ΔH than control samples at pH 7.

**Table 3:** Characteristic enthalpy and peak temperature of the control and ultrasound-treated (20 kHz, 40 kHz, and 20 + 40 kHz) FPIs at pH 3, 7 and 9.

pH	Samples	Peak temperature (℃)	ΔH (J/g)
pH 3	FPI-control	100.76 ± 0.55^b^	1434.00 ± 32.00^a^
	FPI-20	104.94 ± 0.62^a^	1431.50 ± 11.50^a^
	FPI-40	100.18 ± 0.72^b^	1350.50 ± 7.50^b^
	FPI-20 + 40	99.23 ± 0.45^b^	1293.00 ± 7.00^b^
pH 7	FPI-control	100.08 ± 0.5^b^	1318.50 ± 9.50^c^
	FPI-20	102.81 ± 0.46^a^	1629.00 ± 14.00^a^
	FPI-40	100.41 ± 0.45^b^	1581.50 ± 12.50^b^
	FPI-20 + 40	101.83 ± 0.56^ab^	1565.50 ± 7.50^b^
pH 9	FPI-control	99.68 ± 0.45^a^	1311.00 ± 12.00^a^
	FPI-20	96.48 ± 0.62^b^	1068.50 ± 11.50^b^
	FPI-40	96.24 ± 0.52^b^	0921.80 ± 12.40^c^
	FPI-20 + 40	94.69 ± 0.58^b^	0831.60 ± 12.00^d^

Note: Values are given as mean ± standard deviation. The means with different lowercase letters in the same column are significantly different (p < 0.05).

### 3.6 Solubility

**Fig. 6:**
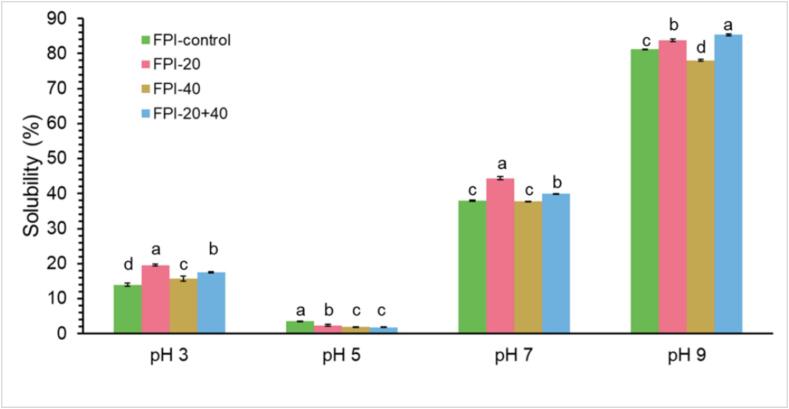
pH-dependent solubility of the control and ultrasound-treated (20 kHz, 40 kHz, and 20 + 40 kHz) FPI.

Protein solubility is an important functional property in the food process. Higher protein solubility is more desirable for the application of the food [45]. Fig. 6 shows the solubility of the control and ultrasound-treated FPI samples at pH 3, 5, 7, and 9. The solubility profile of all FPI samples presented a U-shaped curve with an increase in pH. The lowest value of the protein solubility among the four pHs was at pH 5, in which the solubility was less than 5 %. At pH 5, the net charge of the proteins is close to zero. This could result in the lack of electromagnetic attraction between the protein particulates in this pH condition, which leads to the reduction of the propensity for coagulation [46]. According to the figure, there is a decrease in solubility for ultrasound-treated samples at this pH value.

The solubility increased when the pH value was far away from pH 5. The control FPI showed solubility values of 13.9, 38.1, and 81.3 % at pH 3, 7, and 9, respectively. The ultrasound treatment with 20 kHz and 20 + 40 kHz had a significant improvement in FPI solubility at the pH of 3, 7, and 9 (p < 0.05). Ultrasound treatment generates a cavitation force which could destroy non-covalent interactions (i.e. hydrophobic, electrostatic, hydrogen bonding) in aggregated proteins. The reduction of non-covalent bonds between proteins could result in an increased number of interactions between water and protein molecules, thereby promoting the increase of protein solubility [16]. Other studies have also found that ultrasound treatment increases the solubility of plant proteins [11], [12], [44], [47].

The ultrasound treatment with 40 kHz resulted in different effects on the solubility of FPI at pH 7 and 9 compared to 20 kHz and 20 + 40 kHz. There was no significant difference in solubility at pH 7 between FPI-control and FPI-40 (p < 0.05). FPI-40 decreased in solubility at pH 9 compared to FPI-control. This could be explained by the high solubility of the untreated proteins of over 80 % at pH 9, at which electrostatic reactions mainly affect protein solubility. The different modifications in solubility shown by FPI-20, FPI-40, and FPI-20 + 40 could be attributed to the different degrees of structural change modified by the ultrasound treatment with different intensities. It is possible that more hydrophilic groups are exposed to the surface after ultrasound treatment with 20 kHz and 20 + 40 kHz. FPI-20 and FPI-20 + 40 displayed more obvious improvement in solubility.

## 4 Conclusions

In this research, the ultrasound treatment with different frequencies (20 kHz, 40 kHz, and 20 + 40 kHz) could be an efficient method to modify the structure and properties of faba bean protein, especially at acid and neutral pH values. Ultrasound treatment with different frequencies had no obvious effect on the molecular weight of FPI, whereas it changed the secondary structure of FPI from a more ordered structure to a more disordered structure. The structure change can further lead to the difference in physiochemical properties of FPI. 20 kHz treatment resulted in the most improvement in the surface hydrophobicity at pH 7 and 9, while the surface hydrophobicity of FPI-20 + 40 increased most at pH 3. Such structural change could be beneficial to the application of FPI in the emulsion system. In addition, the solubility and thermal properties of FPI were modified through the ultrasound treatment. The denaturation temperature of FPI-20 significantly increased at pH 3 and 7 (p < 0.05). FPI-20 and FPI-20 + 40 had a significant improvement in FPI solubility at the pH of 3, 7, and 9 (p < 0.05). The higher solubility of FPI could improve its potential to be used as a functional ingredient for many food applications.

Overall, the use of different frequencies (20 kHz, 40 kHz, and 20 + 40 kHz) in ultrasound treatment is an important factor that affects the structure and physiochemical properties of FPIs to varying degrees. Generally, ultrasound treatment using 20 kHz and 20 + 40 kHz frequencies results in more changes to the physiochemical properties of FPI compared to treatment using 40 kHz alone. This study serves as a reference for selecting and optimizing ultrasonic conditions to modify FPIs. Future studies can explore the functional properties of ultrasound-treated FPI and its potential applications in the food industry.

## CRediT authorship contribution statement

**Shuyang Wang:** Writing – original draft, Investigation, Formal analysis, Data curation, Conceptualization. **Song Miao:** Writing – review & editing, Supervision, Resources, Project administration, Funding acquisition, Conceptualization. **Mohammad Hassan Kamani:** Writing – review & editing, Investigation. **Eoin G. Murphy:** Writing – review & editing. **Da-Wen Sun:** Writing – review & editing, Supervision.

## Declaration of competing interest

The authors declare that they have no known competing financial interests or personal relationships that could have appeared to influence the work reported in this paper.
